# The diminution and modulation role of water-soluble gallic acid-carboxymethyl chitosan conjugates against the induced nephrotoxicity with cisplatin

**DOI:** 10.1038/s41598-022-21681-8

**Published:** 2022-11-19

**Authors:** Hani S. Hafez, Ebtesam S. Kotb, Zakaria El-Khayat, Reda F. M. Elshaarawy, Waleed M. Serag

**Affiliations:** 1 Zoology Department, Faculty of Science, Suez University, Suez, 43533 Egypt; 2 Chemistry Department, Faculty of Science, Suez University, Suez, 43533 Egypt; 3 Medical Biochemistry Department, National Research Center Egypt, Giza, Egypt

**Keywords:** Biochemistry, Chemical biology, Chemistry, Materials science

## Abstract

The toxicity of cisplatin (CDDP) toward the renal tubules and its severe effects on the proximal tubules limits its further use in cancer therapy. The current study was undertaken to evaluate the protective effects of gallic acid-grafted O-carboxymethyl chitosan (GA@CMCS) against nephrotoxicity induced by CDDP in rats. Renal injury was assessed in the GA@CMCS/CDDP-treated rats using kidney injury molecule-1 (KIM-1). Moreover, the levels of reduced glutathione (GSH), malondialdehyde (MDA), and nitric oxide (NO) were measured. The comet assay was performed to measure the DNA damage. The renoprotective activity of GA@CMCS was supported by histo- and immuno-pathological studies of the kidney. GA@CMCS significantly normalized the increases in kidney homogenate of KIM-1, MDA, and NO-induced by CDDP and significantly increased GSH as compared with the CDDP group. GA@CMCS also significantly protects rat kidneys from CDDP-induced histo- and immuno-pathological changes. Both biochemical findings and histo- and immuno-pathological evidence showed the renoprotective potential of GA@CMCS against CDDP-induced oxidative stress, inflammation, and renal dysfunction in rats. In conclusion, GA@CMCS has been shown to mitigate the nephrotoxicity impact of CDDP in cancer therapy.

## Introduction

Although the rapid progress in cancer treatment has changed the oncology landscape of treatment with decreased tumor progression and increased survival rates^1^, cancer patients still experience severe side effects during their chemotherapy courses, such as nephrotoxicity, neurotoxicity, ototoxicity, cardiotoxicity, hepatotoxicity, hemotoxicity, and gastrointestinal toxicity^2^. Nephrotoxicity is remaining a significant complication limiting factor that lessens the efficacy of many drugs^1^. Nephrotoxicity produces harmful effects in the body and increases the body's ion levels, serum creatinine, serum total protein, serum urea, and blood urea nitrogen (BUN)^3^. According to the National Institutes of Health, there are currently over 273,000 cancer-related clinical trials underway around the world, with the majority of the tested drugs being Pt-based treatments^4^.

Although cisplatin (cis-diamminedichloroplatinum(II), CDDP) has been a mainstay in cancer therapy, its use is limited by two issues: the development of resistance to CDDP and the severe side effects in normal tissues caused by the high tendency to accumulate renal toxin nephrotoxins in the proximal tubules, which causes severe damage^5^. Notably, CDDP undergoes hydrolysis, in which one or two chloride ions are replaced by water generating positively charged electrophiles [Pt(NH_3_)_2_(H_2_O)_2_]^2 +^ and [Pt(NH_3_)_2_Cl(H_2_O)]^+^ that are highly reactive with nucleophilic sites (Fig. S1, ESM S1)^5,6^. These positively charged platinum complex ions are more harmful to kidney cells than CDDP because they can bind to nuclear components like DNA, RNA, and proteins to cause renal cell necrosis^7^. The ultimate reno-protection approaches should not only protect the kidneys but also enhance CDDP’s anticancer effectiveness in chemotherapy and lessen their by-products toxicity^8^. Antioxidants can preserve living tissue by either preventing the activation of oxygen for highly reactive substrates or scavenging oxidant species before they interact with biological targets^9^. Gallic acid (GA) is a natural polyphenolic antioxidant widely found in many natural products^10,11^. It has been found that GA and its derivatives have a wide variety of favorable benefits in the prevention and/or treatment of several illnesses. Their good safety and stability profiles also make them viable candidates for the introduction of dietary supplements^12^. GA's protective function is due to being a strong natural antioxidant that can inhibit reactive oxygen species (ROS), e.g., hydrogen peroxide, superoxide anions, hypochlorous acid, and hydroxyl radicals, thereby preventing oxidative stress in tissues^13,14^. GA has been reported to avert many disorders, including cardiovascular disease, cancer, inflammation, infection, diabetes, and neurological impairments^15^.

Several difficulties have been encountered in using natural antioxidants in pharmaceutical, biomedical, and food products, including volatility, instability, and oxidative conditions. Therefore, it is important to immobilize the natural antioxidants onto the surface of stable solid supports such as biopolymers and inorganic materials to overcome the aforementioned obstacles^16^. Chitosan (CS) as a highly used polysaccharide has received a lot of attention because of its biodegradability, biocompatibility, non-antigenicity, and non-toxicity^17^. Additionally, it has been proven to hold a wide range of specific properties such as antimicrobial, antitumor, anti-inflammatory, antioxidant, and anti-hypercholesterolemic roles. Many disciplines, including biomedicine, pharmaceuticals, water treatment, functional membranes, and the food industry, have benefited amazing potential for CS^17,18^. Noteworthy, grafting phenolic compounds has been shown to give polymers new qualities, such as improved mechanical and physiological properties and variations in solubility, antioxidant and emulsifying capabilities. In addition, the bioactivities of phenolic compounds grafted onto polymers were higher than those of the free phenolic compounds in question^19^. In this context, recently, more attention has been driven to biomacromolecules functionalization such as CS or any other derivative thereof with naturally occurring flavonoids such as gallic acid or caffeic acid, or catechins^16,20^. Because of the distinct properties of CS and GA, as well as our ongoing pursuit of new highly effective chemotherapeutic candidates^21–23^. The present study aimed to investigate the biomedical and renoprotective of gallic acid-grafted O-carboxymethyl chitosan, additionally, to study their anti-inflammatory and antioxidant activity against the CDDP by-products in vivo*.*

## Materials and methods

Details for chemicals and solvents used in this work along with their suppliers were provided in the electronic supplementary material (ESM S1). Also, the extraction of CS from crab shells and its carboxymethylation, to prepare O-carboxymethyl chitosan (CMCS), was described in the ESM S1. In addition, instrumentation and different analytical techniques used for the full characterization of all synthesized materials were described in ESM S1.

### Preparation of gallic acid-grafted O-carboxymethyl chitosan (GA@CMCS)

The GA@CMCS was prepared following the previously described protocol^24^ with a slight modification. Briefly, solution **A** was prepared by dissolving 1.25 g of carb CS (6.12 mol NH_2_) in a mixed-solvents system of deionized water/methanol (1/2 v/v, 30 ml), then the obtained solution was adjusted to pH 6.8 with trimethylamine. In solution **B**, Gallic acid (1.12 g; 6.58 mmol) was dissolved in 10 ml of methanol and mixed with a solution of dicyclohexylcarbodiimide (DCC) (0.63 g; 3.28 mmol) in methanol (10 ml) under stirring. After 1 h, solution **B** was gradually added to solution **A** while stirring (150 rpm) in an ice bath for 1 h. Thereafter, the reaction mixture was removed from the ice and stirred overnight at room temperature. Then, the content was filtered to remove dicyclohexylurea (DCU) and the filtrate was kept at 2 °C overnight. Then, the reaction mixture was diluted with 90 ml of diethyl ether to precipitate the desired product. The isolated product was collected by filtration and then dissolved in deionized water (20 ml) and dialyzed against deionized water using dialysis tubing of a molecular weight cut-off (12,000–14,000 Da) for 48 h with 6 times deionized water changes to ensure the complete removal of un-reacted GA. Finally, the resulting solution was lyophilized at room temperature to obtain water-soluble GA@CMCS.

### Experimental design

Sixty-four Sprague-Dawley male white albino rats (1–2 months old, 120–150 g weight) were obtained from the Animal House of National Research Center, Dokki, Giza, Egypt. The rats were housed in groups (4/cage) until surgery, after which they were singly housed. The rats were kept with free access to a standard pellet animal diet and tap water under controlled temperature (24 ± 1 °C) and 12-h light–dark cycle (lights on 6:30 a.m.), and testing was carried out during the light phase. All animal experiments were performed in accordance with relevant guidelines and regulations according to The ARRIVE guidelines 2.0: Updated guidelines for reporting animal research^25^ and were approved by the National Hepatology & Tropical Medicine Research Institute's ethics committee (NHTMRI) code: 6A-2021. Six rats were used in each group, and all were anesthetized with an intraperitoneal dose of 80 mg/kg sodium pentobarbital followed by normal saline for transcardial perfusion. Kidneys were extracted and were classified for the different tests of histological and immunohistological section staining. All efforts were made to minimize animal pain, suffering, or discomfort as well as the number of rats used in all experiments. Animal's grouping was done as follows.

- Group 1 (normal control group): without treatment.
- Group 2 (GA group): healthy rats were intraperitoneal injection (i.p.) injected with GA at a dose of 40 mg/kg body weight/day for 14 days; the dose was chosen based on the previous study according to Akomolafe et al.^26^.
- Group 3 (CMCS group): healthy rats were i.p. injected with CMCS (178.625 mg/kg body weight/day) for 14 days; the dose was modified from a previous study by Ibrahim, et al.^27^ which stated that LD_50_ of carboxymethyl CS was 3.5725 g/kg body weight in mice.
- Group 4 (GA@CMCS group): healthy rats were i.p. injected with GA@CMCS at a dose of 323.3 mg/kg body weight/day for 14 days.
- Group 5 (CDDP group): healthy rats were i.p. injected with CDDP (12 mg/kg body weight) once on the tenth day during the experimental period to induce nephrotoxicity; the dose was selected from a previous study by Dizaye^28^.
- Group 6 (CDDP + GA) treated: rats have received GA in a dose of 40 mg/kg body weight/day, i.p. for 14 days with a single dose of CDDP (12 mg/kg body weight, i.p.) on the tenth day. CDDP was administered an hour after receiving the dose of GA^29^.
- Group 7 (CDDP + CMCS) treated: the rats have received a 14-day treatment of CMCS in a dose of 178.625 mg/kg body weight/day, i.p. with a single dose of CDDP (12 mg/kg body weight, i.p.) on the tenth day. CDDP was administered an hour after receiving the dose of CMCS.
- Group 8 (CDDP + GA@CMCS) treated: the rats received GA@CMLMWC in a dose of 323.3 mg/kg body weight/day, i.p. for 14 days with a single dose of CDDP (12 mg/kg body weight, i.p.) on the tenth day. CDDP was administered an hour after receiving the dose of GA@CMCS.

All treatments were administered daily for 14 days except CDDP (a single i.p. injection on the tenth day of the treatment schedule) using a dosing schedule modified from the previous study by Kamel et al.^30^. Five days post-CDDP administration^31^, rats were weighed and 3 ml of blood from each rat was withdrawn from the retro-orbital venous plexus of the eye using capillary tubes and collected in ethylenediaminetetraacetic acid (EDTA) tube for collecting whole blood for the comet assay. The rats were then sacrificed, and the two kidneys were quickly removed, washed with cold saline solution (0.9% NaCl), and weighed. Then one kidney was homogenized as per a procedure described in the literature^32^ and prepared for measuring kidney injury molecule-1 (KIM-1), malondialdehyde (MDA), and reduced glutathione (GSH), and nitric oxide (NO) parameters. The other kidney was fixed in a 10% neutral buffered formalin solution for histopathological examination and immunohistochemical studies of cyclooxygenase-2 (COX-2), caspase 3, and sodium–potassium adenosine triphosphatase (Na^+^/K^+^-ATPase) according to the manufacturer’s instructions.

### Body and kidneys weight changes

Changes in the body and kidney weights of rats from all groups were observed and renal index (%) was calculated according to the formula: (kidney weight/total body weight) × 100^33^.

### Antioxidant assessments

#### Determination of tissue lipid peroxidation

The lipid peroxides content in kidney homogenate was determined by estimation of MDA level using the method described by Ohkawa et al.^34^.

#### Determination of tissue GSH levels

Determination of GSH content was measured according to the colorimetric method^35^.

### Assessment of nitrosative stress

The renal nitrite levels (NO) were determined as per the procedure described by Gonzalez-Barrios et al.^36^ with a slight modification of the Griess reagent system as stated by Sahu et al.^37^.

### Estimation of an immunological marker for kidney damage

Quantitative determination of rat KIM-1 by enzyme-linked immunosorbent assay (ELISA) in kidney tissue homogenate was determined according to the manufacturer’s instruction (SunLong Biotech Co. Ltd., China, Catalogue Number: SL0433Ra).

### Assessment of DNA damage (Comet assay)

The comet test was carried out following the protocol reported by Singh et al.^38^, which was slightly modified by Blasiak et al.^39^. An inverted fluorescent microscope (IX70; Olympus, Tokyo, Japan) with a 549 nm excitation filter and a 590 nm barrier filter, coupled to a video camera (Olympus), was used to analyze the slides at 40× magnification. Damaged cells took on the appearance of a comet, with a brilliantly fluorescent head and a long, brightly fluorescent tail that were separated from one another during electrophoresis. Images were analyzed using Image J (IJ 1.46r) to count damaged cells with their configuration.

### Histopathological assessment

Kidney tissue was immediately removed and fixed in 10% neutral buffered formalin for one day before being dehydrated in increasing alcohol concentrations, purified with xylene, and then immobilized in paraffin. Afterward, paraffin blocks were cut into tissue sections (5 µm) and stained with Hematoxylin–Eosin (H&E) as previously described by Moradi et al.^40^.

### Immunohistochemistry (IHC) analysis

Sections were successively treated for 30 min with 0.3% H_2_O_2_ in PBS and 10% goat serum in PBST. After that, the sections were incubated overnight at room temperature with rabbit anti-COX2 antibodies (1:50 μg/ml Leader in Biomolecular Solutions for Life Science, Catalog No.: A1253), caspases-3 (1:50 μg/ml Leader in Biomolecular Solutions for Life Science, Catalog No.: A11953), ATPase, (Na(+) K(+)) alpha subunit (a5, 2–5 μg/ml, Developmental Studies Hybridoma Bank (DSHB), Iowa University, USA). Finally, these sections were treated with biotinylated goat anti-mouse IgG (1:200) (Vector Laboratories, Catalog No.: BA-9200-1.5) followed by streptavidin peroxidase complex (1:200) (Vector Laboratories, Catalog No.: SA-5004-1).

### Statistical analysis

Kidney sections were examined and proteins expression has been quantified using Image J software (https://imagej.nih.gov/ij/) and processed in Prism 7 software (GraphPad, San Diego, CA, United States) (https://en.freedownloadmanager.org/users-choice/Graphpad_Prism_7_Free_Download.html), all data have been presented as mean ± standard errors (SE) for each protein distribution in the different areas of the kidney tissue. The IBM SPSS software package version 20.0 (Armonk, NY: IBM Corp) was used to analyze the data that was entered into the computer. Quantitative data were described using mean and SE. The significance of the obtained results was judged at the 5% level. Statistical comparisons between experimental groups were performed using a one-way analysis of variance (ANOVA) test, followed by Tukey’s multiple comparison post hoc test for pairwise comparisons. Differences with values of p < 0.05 were considered statistically significant.

## Results

### Preparation of GA@CMCS conjugate

**Figure 1:**
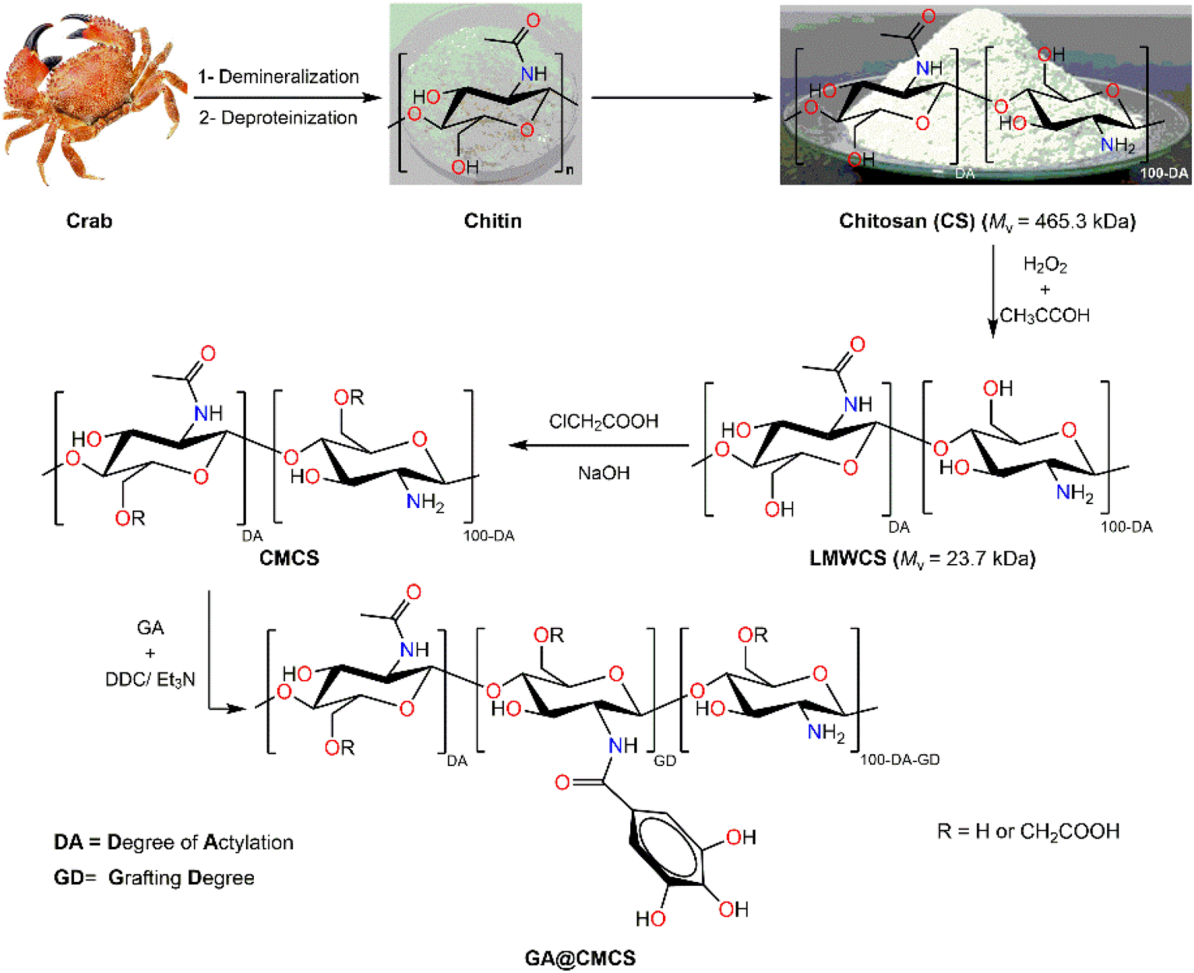
Schematic diagram for the extraction of crab chitosan, its partial degradation, carboxymethylation, and grafting of GA for the preparation of GA@CMCS conjugate.

Initially, CS was extracted from crab shells using our routine protocol that involved demineralization, deproteinization, and deacetylation processes^41^. The obtained crab CS was subjected to partial degradation mediated by hydrogen peroxide to low-molecular-weight chitosan (LMWCS) which was submitted to the carboxymethylation reaction for the C6-OH group using chloroacetic acid at ambient conditions to yield O-carboxymethyl low molecular weight chitosan (CMCS)^42^ (see Fig. 1). Eventually, the amide coupling reaction between the amino group of CMCS and carboxylic group of GA has been carried out using DCC as a coupling agent to graft the surface of CMCS with GA, aiming fabrication of GA@CMCS conjugate.

### Structural characterization of GA@CMCS conjugate

The synthesized materials were obtained in excellent yields and structurally characterized using the elemental and spectral analyses (FTIR, UV–Vis, NMR (^1^H, ^13^C)).

Viscosity average molecular weight (M_v_), degree of deacetylation (DD), degree of polymerization (DP_v_), carboxymethylation degree (CD), and grafting degree (GD). The results of these experiments were collected in Table 1.

**Table 1:** Viscometric average molecular weights (*M*_v_), degree of polymerization (*DP*_v_), degrees of deacetylation (DD), carboxymethylation degree (CD), and grafting degree (GD) of new materials.

Sample	*M*_*v*_^a^ (kDa)	*DP*_*v*_^a^	DD%^b^			EA Calcd (Found) (%)			CD%^c^	GD%^d^
			^1^H-NMR	FTIR	EA	C	H	N		
CS	463.8	2790	87.3	83.5	86.4	45.14 (44.99)	6.81 (6.87)	8.39 (8.29)	–	–
LMWCS	24.5	143	88.9	84.7	87.7	45.10 (45.03)	6.82 (6.86)	8.42 (8.38)	–	–
CMCS	33.33	142.4	79.5	78.5	74.2	42.73 (42.68)	6.27 (6.28)	6.14 (6.03)	67.9	–
GA@CMCS	31.38	142.2	–	–	–	44.84 (44.35)	5.66 (5.67)	5.11 (5.02)	–	41.3

^a^*M*_v_ and *DP*_v_ were quantified based on the values of the intrinsic viscosities of their respective solutions in 0.10 M NaCl at 25 °C (See ESM S1).

^b^DD values were calculated from the EA and the spectroscopic data (IR and ^1^H-NMR) (See ESM S1).

^c^CD was estimated using acid–base titration (See ESM S1).

^d^GD was estimated using Folin–Ciocalteau method^43^ (See ESM S1). The GD value was 41.3% (i.e. 413 mg of GAs per 1 g of CMCS).

### Fourier-transform infrared spectroscopy (FTIR) analysis

**Figure 2:**
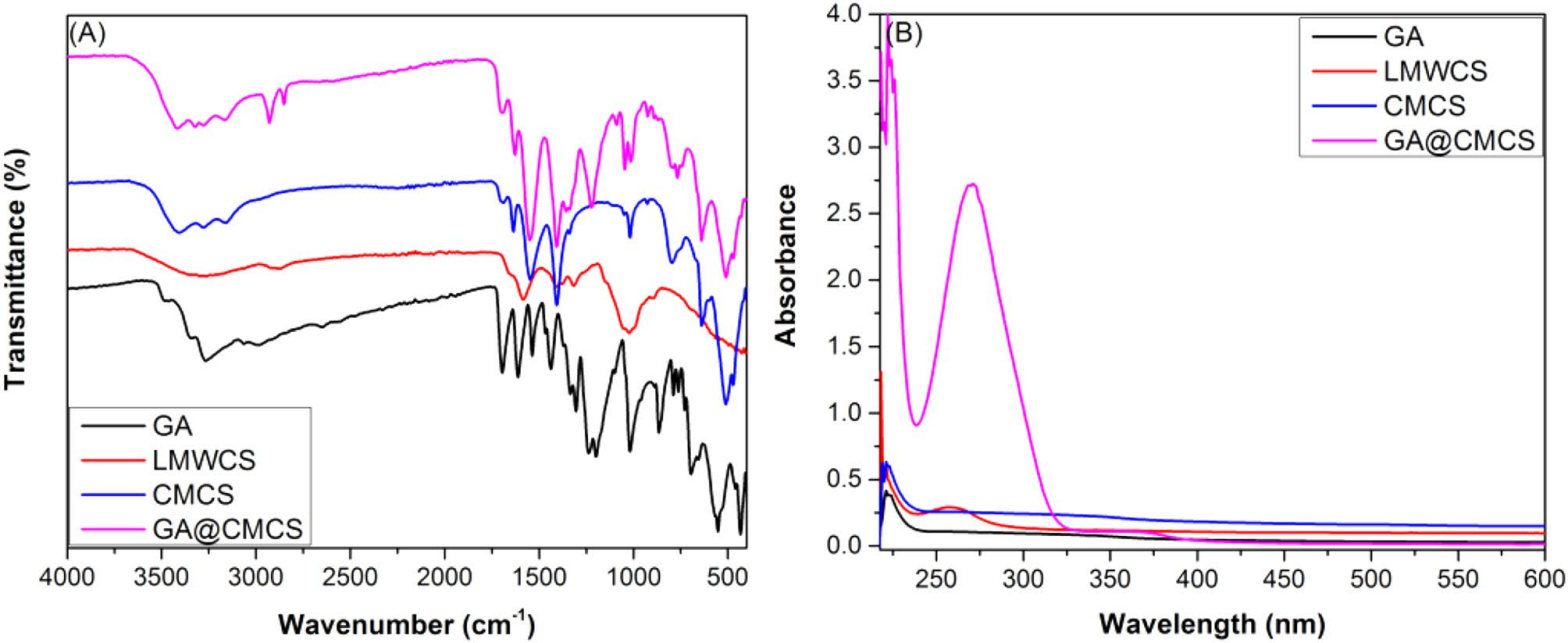
(**A**) Collective FTIR spectra of GA, LMWCS, CMCS, and the conjugate (GA@CMCS) for comparison of their respective characteristic vibration bands. (**B**) UV–Vis spectra of the GA, LMWCS, CMCS, and (GA@CMCS) in aqueous acetic acid (0.5%, v/v) (0.5 mg/mL).

The FTIR spectra of GA, LMWCS, CMCS, and the conjugate (GA@CMCS) were depicted in Fig. 2A. In the spectrum of GA, it can observe the characteristic bands at 3461 and 3266 cm^−1^ (phenolic O–H), 1370 and 1272 cm^−1^ (in-plane bending and vibration O–H and Aryl-O), 1696 cm^−1^ (C=O of the carboxyl group), 3061, 1612 and 1537 cm^−1^ (Ar‒H and C=C of a benzene ring). The FTIR spectrum of *O*-CMCS displayed characteristic IR bands centered at 3384, 3278, 2904 cm^−1^ (alcoholic O–H/1° amine N–H and aliphatic C–H), 1596 and 1417 cm^−1^ (COO^−^ asymmetrical and symmetrical stretching vibrations), 1596 cm^−1^ (N–H bending of NH_2_), 1062 cm^−1^ (primary hydroxyl group (C6‒OH)).

### UV–Vis spectra

Figure 2B shows the UV–Vis spectra of GA, CMCS, and GA@CMCS conjugate.

The spectrum of GA shows a peak at 264.00 nm (π → π* transition in the benzene ring). On the other hand, the UV–Vis spectrum of LMWCS displayed an intense UV peak at 225 nm and a broad absorption peak at 265.50 nm in the UV region. Noteworthy, the chloromethylation of CS results in the almost complete disappearance of the 225 nm peak and the emergence of a new abroad band at 344.50 nm for CMCS (n → π* transition of the carboxyl group)^44^.

### Body and kidneys weight changes results

**Figure 3:**
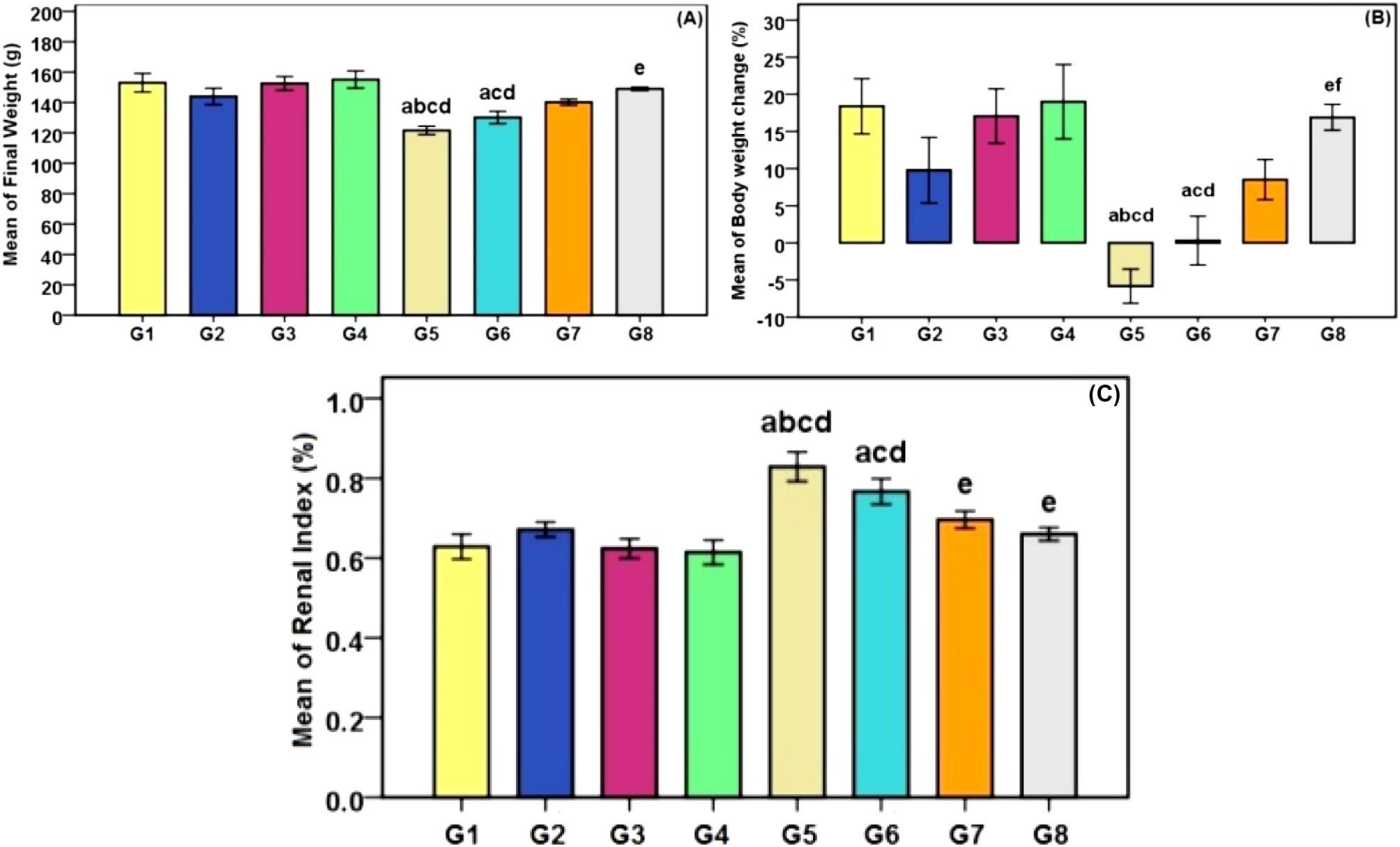
The effect of different treatments in CDDP-induced nephrotoxicity on (**A**) kidney weight of rats (**B**) body weight change (%) of rats, and (**C**) renal index of rats in experimental groups. G1, Control; G2, GA; G3, CMCS; G4, GA@CMCS; G5, CDDP; G6, CDDP + GA; G7, CDDP + CMCS; G8, CDDP + GA@CMCS. Values are means ± SE (*n* = 8). Data were analyzed by a one-way ANOVA test followed by Tukey’s post hoc test for multiple comparisons. ^a^p < 0.001 versus control group (G1), ^b^p < 0.001 vs. GA (G2), ^c^p < 0.001 vs. CMLMWC group (G3), ^d^ p < 0.001 vs. GA@CMCS group (G4). ^e^p < 0.001 versus the CDDP group (G5), ^f^ p < 0.001 versus the (CDDP + GA) group (G6).

The CDDP-treated group showed a significant decrease in the final body weight of the rats as compared with the control group, GA group, CMCS group, and GA@CMCS group (p < 0.001) (See Fig. 3). Also, the body weights of rats in the (CDDP + GA) group significantly decreased in comparison with the control group, CMCS group, and GA@CMCS group (p < 0.001), whereas in the (CDDP + GA@CMCS) group, the treatment with GA@CMCS reversed body weight loss in the present study Fig. 3A. The body weight change (%) of rats in the CDDP group significantly decreased in comparison with the control group, GA group, CMCS group, and GA@CMCS group.

Also, the body weight change (%) of rats in the (CDDP + GA) group significantly decreased in comparison with the control group, CMCS group, and GA@CMCS group, whereas in the (CDDP + GA@CMCS) group, the treatment with GA@CMCS restored body weight loss compared with the CDDP group or (CDDP + GA) group Fig. 3B. The renal index in the CDDP group was significantly higher than that in the control group, GA group, CMCS group, and GA@CMCS group. Also, the renal index in the (CDDP + GA) group was significantly higher than that in the control group, CMCS group, and GA@CMCS group, whereas in the (CDDP + CMCS) group and (CDDP + GA@CMCS) group, the treatment with CMCS and GA@CMCS respectively decreased the index significantly compared with the CDDP group Fig. 3C.

### Antioxidant indices assessment

#### Malondialdehyde (MDA)

The current research showed that MDA levels in the kidney homogenates of rats administered intraperitoneal CDDP with 12 mg/kg body weight have been significantly upregulated with p < 0.001 in comparison to the control group (Table 2). Moreover, our study demonstrated that the rats treated with GA, CMCS, and GA@CMCS before and after administration of CDDP significantly mitigated the CDDP-induced nephrotoxicity and oxidative damage.

**Table 2:** Comparison of oxidative stress indicators and various parameters in renal tissues between different study groups.

	MDA (nmole MDA/g tissue)	NO (µmoles/g tissue)	GSH (µmole GSH/g tissue)
Group 1 (control)	52.35 ± 10.39	29.97 ± 3.03	5.27 ± 0.35
Group 2 (GA)	59.29 ± 8.37	25.03 ± 1.74	4.93 ± 0.48
Group 3 (CMCS)	56.09 ± 13.64	25.45 ± 6.17	5.01 ± 0.50
Group 4 (GA@CMCS)	58.61 ± 7.33	31.98 ± 2.26	5.20 ± 0.70
Group 5 (CDDP)	165.9^abcd^ ± 21.28	50.87^abcd^ ± 5.08	2.43^abcd^ ± 0.38
Group 6 (CDDP + GA)	100.4^e^ ± 7.15	37.59 ± 4.96	3.19 ± 0.42
Group 7 (CDDP + CMCS)	83.33^e^ ± 8.36	34.02 ± 2.17	4.12 ± 0.13
Group8(CDDP + GA@CMCS)	69.71^e^ ± 7.51	27.10^e^ ± 1.22	4.42^e^ ± 0.41
F	10.444***	5.301***	5.014***
p	< 0.001***	< 0.001***	< 0.001***

Data were expressed by using (Mean ± SE.), (*n* = 8). F: F for the ANOVA test, pairwise comparison between every 2 groups was done using the Post Hoc Test (Tukey). p: p-value for comparing the different studied groups. ^a^Significant with Group 1 (Control group). ^b^Significant with Group 2 (GA group). ^c^Significant with Group 3 (CMCS group). ^d^Significant with Group 4 (GA@CMCS group). ^e^Significant with Group 5 (CDDP group). ***Statistically high significance at p ≤ 0.001.

#### Glutathione (GSH)

On one side, GSH levels in the CDDP group's kidney homogenate were significantly reduced compared to the control group (p < 0.001) (Table 2). On the other side, the treatment with GA@CMCS revealed the most ameliorated state for the upregulation of the GSH among CDDP administrated rats.

#### Nitric oxide (NO)

The present research revealed that NO levels in the CDDP group were much higher than in the control group, while GA@CMCS treating rats ameliorated CDDP administration toxicity with highly significantly reduced nephrotoxicity and oxidative damage levels. Although treatment with GA or CMCS before and after CDDP administration lowered NO levels, the differences were not significant in comparison to the CDDP group. Whereas, normal rats given GA, CMCS, or GA@CMCS alone for 14 days did not reveal a significant change in NO levels as compared to rats of the control group (Table 2).

### Immunological marker for kidney damage and serum markers of renal injury

**Figure 4:**
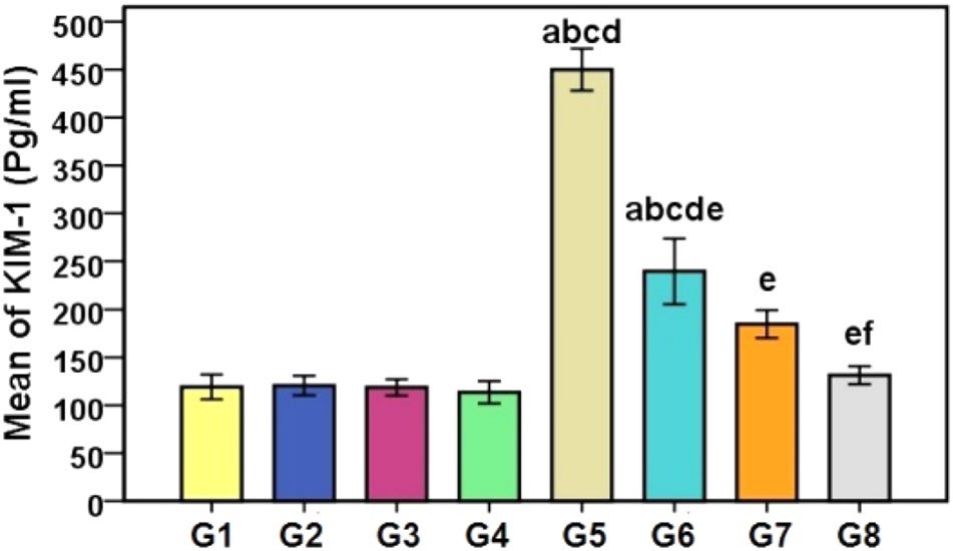
The effect of different treatments on KIM-1 levels in CDDP-induced nephrotoxicity in all the studied groups. Values are means ± SE (*n* = 8). Data were analyzed by a one-way ANOVA test followed by Tukey’s post hoc test for multiple comparisons. ^a^p < 0.001 versus control group (G1), ^b^p < 0.001 vs. GA (G2), ^c^p < 0.001 vs. CMCS group (G3), ^d^p < 0.001 vs. GA@CMCS group (G4). ^e^p < 0.001 versus the CDDP group (G5), ^f^p < 0.001 versus the (CDDP + GA) group (G6).

In the current study, KIM-1 levels were considerably higher in the CDDP group and the (CDDP + GA) group in comparison to the control group. However, rats treated with GA, CMCS, or GA@CMCS before and after CDDP administration significantly decreased KIM-1 levels compared with the CDDP group. In addition, KIM-1 levels were considerably lower in the (CDDP + GA@CMCS) group compared with the (CDDP + GA) group as shown in Fig. 4.

### Comet Assay analysis

**Figure 5:**
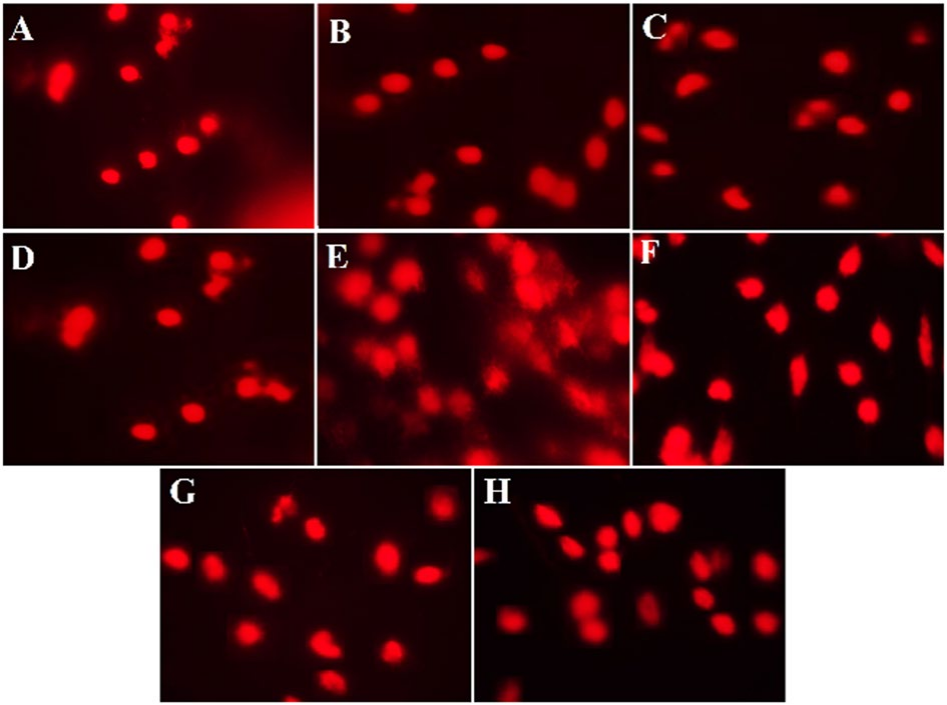
Comet assay showing the percentage of DNA damage in rats subjected to different treatments in CDDP-induced nephrotoxicity. (**A**) the control group showed a small percentage of DNA damage (2%—5%). (**B**) the GA group showed (6–15%) DNA damage. (**C**) the CMCS group showed (7–13%) DNA damage. (**D**) the GA@CMCS group showed (5–11%) DNA damage. (**E**) high DNA damage (70–80%) was observed in the CDDP group compared with the control group. (**F**) the (CDDP + GA) group revealed reduced DNA damage compared with the CDDP group (30–40%). (**G**) the (CDDP + CMCS) group revealed reduced DNA damage compared with the CDDP group (20–30%). (**H**) the (CDDP + GA@CMCS) group revealed reduced DNA damage compared with the CDDP group (27–30%).

The present study revealed high DNA damage in the CDDP-treated group (70 percent -80 percent) (Fig. 5) compared to the control group (2–5%). Consistently, our findings demonstrated that rats given GA, CMCS, or GA@CMCS before and after CDDP injection had less DNA damage compared to the CDDP group.

### Histopathological analysis

**Figure 6:**
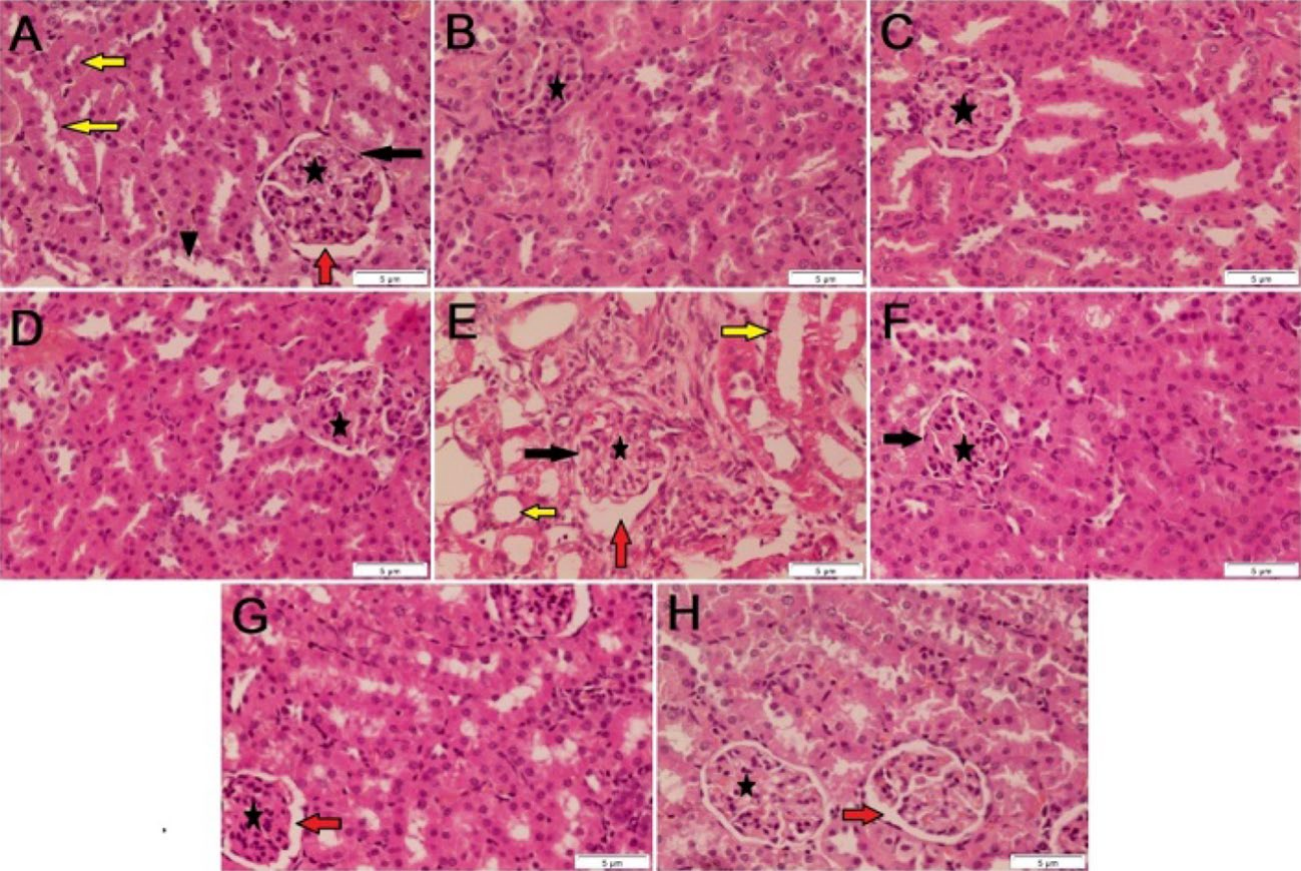
Histological evaluations of kidney sections in all studied groups. The control group (**A**) showed a normal structure of the renal corpuscle (asterisk) and renal tubules, proximal convoluted tubules (yellow arrow), and distal convoluted tubules (arrowhead). Notice the glomerulus (asterisk), urinary space (red arrow), and Bowman's capsule (black arrow). The GA group (**B**), the CMCS group (**C**), and the GA@CMCS group (**D**) all show a normal structure of the renal corpuscle (asterisk) and renal tubules. The CDDP group (**E**) showed the cortical tubules have become shrunken and atrophic and have relatively expanded interstitial spaces. Fibrosis and atrophy of the glomerulus are seen. Notice the glomerular basement membrane is diffusely and fairly uniformly thickened. The (CDDP + GA) group (**F**), the (CDDP + CMCS) group (**G**), and the (CDDP + GA@CMCS) group (**H**) show the structure of the renal corpuscle (asterisk) and renal tubules appear more or less normal. (H & E stain, scale bar: 5 µm).

CDDP administration produces histological kidney abnormalities such as glomerular atrophy, tubule cell fragments, and enlarged epithelial cells in the proximal and distal convoluted tubules, according to our findings. Taking into consideration, treatment of rats with GA*,* CMCS, or GA@CMCS alone did not show any significant histopathological modifications in the kidney compared with the control group. In addition, treatment of the rats with GA*,* CMCS, and GA@CMCS before and after CDDP administration indicated significant improvement in proximal and distal convoluted tubules and glomerular atrophy, with the shape of the renal corpuscle and renal tubules appearing more or less normal (Fig. 6).

Blind pathologist have carried out the histopathological examination of the renal tubule and estimated the semi-quantitative grading system scale: normal = 0, mild ≤ 25%, moderate = 25–50% and severe ≥ 50% of affected area. As follows:

The control group (A) showed a normal structure of the renal corpuscle (asterisk) and renal tubules, proximal convoluted tubules (yellow arrow), and distal convoluted tubules (arrowhead) with score = 0. Notice the glomerulus (asterisk), urinary space (red arrow), and Bowman's capsule (black arrow). The GA group (B), the CMCS group (C), and the GA@CMCS group (D) all show a normal structure of the renal corpuscle (asterisk) and renal tubules with score = 0. The CDDP group (E) showed the cortical tubules have become shrunken and atrophic and have relatively expanded interstitial spaces. Fibrosis and atrophy of the glomerulus are seen. Notice the glomerular basement membrane is diffusely and fairly uniformly thickened with score > 50%. The (CDDP + GA) group (F), the (CDDP + CMCS) group (G), and the (CDDP + GA@CMCS) group (H) show the structure of the renal corpuscle (asterisk) and renal tubules appearing more or less like normal with score ≤ 25%. (H & E stain, scale bar: 5 µm).

### Immunohistochemistry analysis

#### Cyclooxygenase-2 (COX-2)

**Figure 7:**
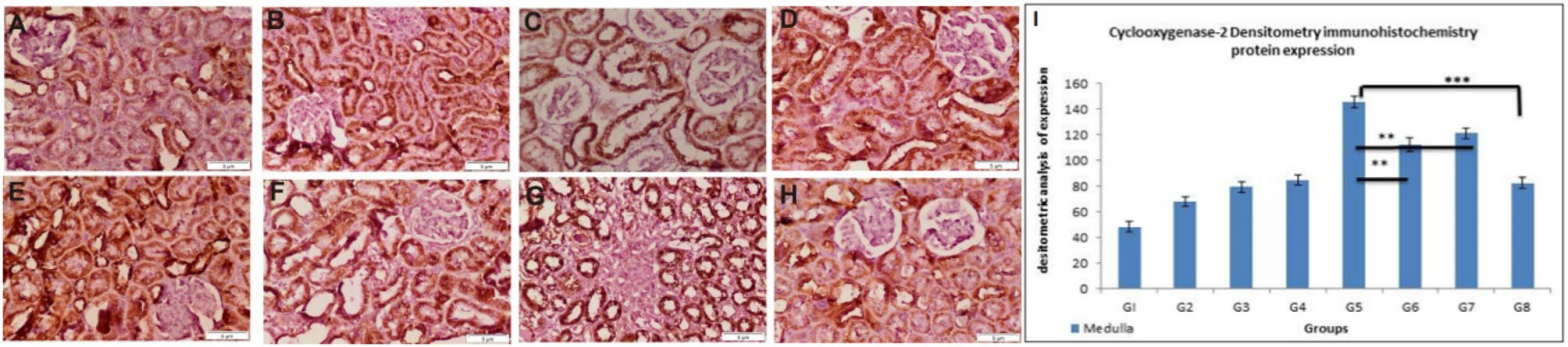
The effect of different treatments on COX-2 immunoreactivity and densitometry in CDDP-induced nephrotoxicity in all the studied groups. (**A**–**H**) COX-2 immunoreactivity evaluations in the kidney of rats. The control group (**A**), the GA group (**B**), the CMCS group (**C**), and the GA@CMCS group (**D**) showed a pale stain in the proximal tubular cells and a pale stain in the distal convoluted tubules and glomeruli. The CDDP group (**E**) showed intense COX-2 immunoreactivity in the proximal tubular cells as compared with the control. The (CDDP + GA) group (**F**), and the (CDDP + CMCS) group (**G**) showed moderately decreased COX-2 immunoreactivity in the proximal convoluted tubules as compared with the CDDP group. The (CDDP + GA@CMCS) group (**H**) showed a pale stain in the proximal convoluted tubules as compared with the CDDP group. (COX-2 expression, scale bar: 5 µm). All data have been presented as mean ± SE. **p < 0.01, and ***p < 0.001. (**I**) COX-2 densitometry immunohistochemistry expression analysis in the medulla in all studied groups.

We discovered that a single dose of CDDP (12 mg/kg body weight, i.p.) induces a significant increase in COX-2 immunoreactivity in the proximal tubular cells as compared to the control group (Fig. 7A–H). Figure 7I showed that treating rats with GA or CMCS for ten days before CDDP injection and another 4 days after CDDP injection significantly reduced COX-2 densitometry immunohistochemistry expression when compared to the CDDP group (p < 0.01) and that the COX-2 densitometry immunohistochemistry expression was highly significant decreased in the (CDDP + GA@CMCS) group when compared to the CDDP group (p < 0.001). In comparison to the control group, we identified no significant immunohistochemistry changes in the kidneys of rats treated solely with GA, CMCS, or GA@CMCS.

#### Caspase-3

**Figure 8:**
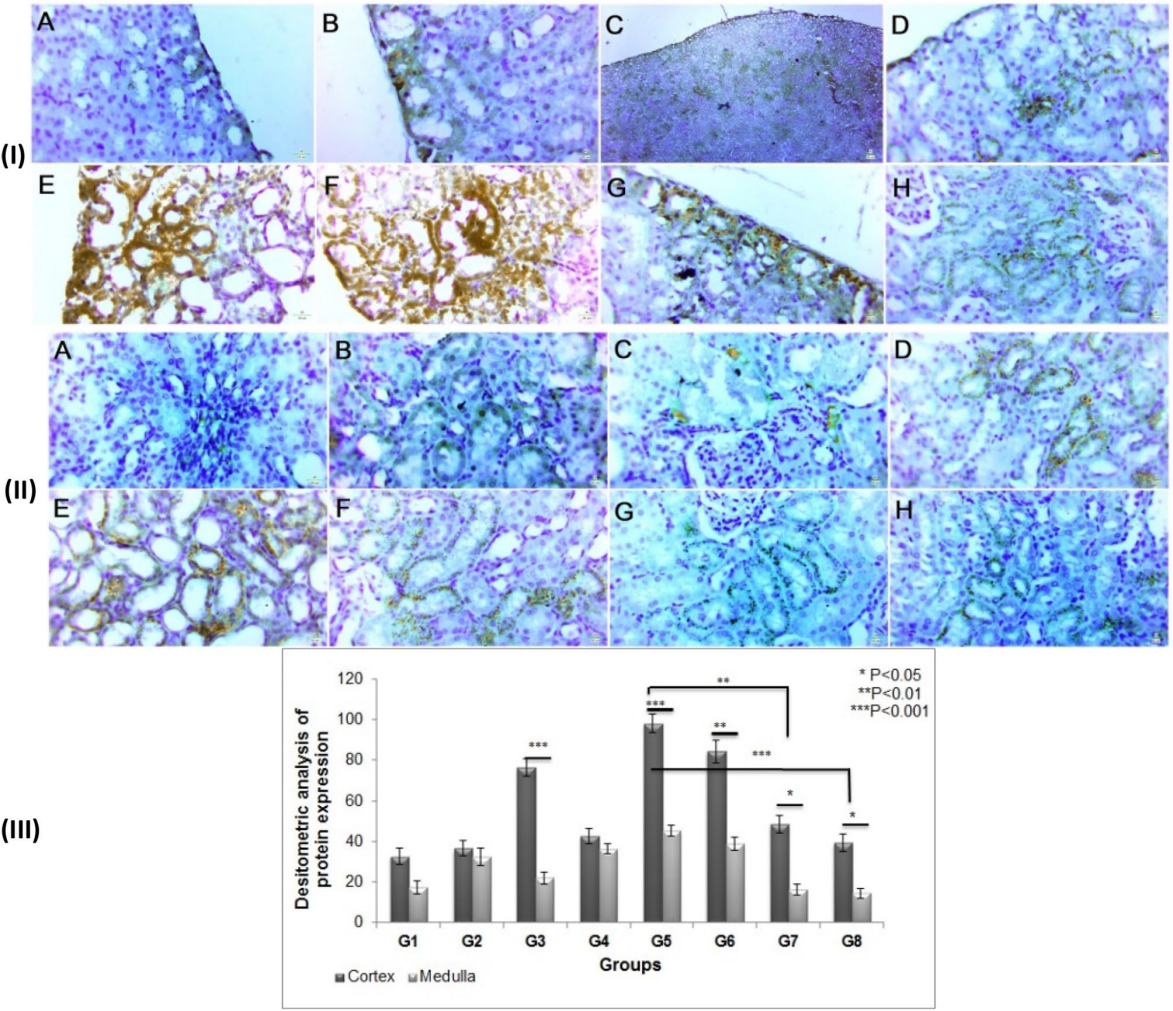
The effect of different treatments on caspase-3 immunoreactivity and densitometry in CDDP-induced nephrotoxicity in all the studied groups. Caspase-3 immunoreactivity evaluations in the cortex (**I**) and the medulla (**II**) of the kidney of rats. (**III**) caspase-3 densitometry immunohistochemistry expression analysis in both cortex and medulla in all studied groups. Caspase-3 expression using immunohistochemical staining in kidney sections: (**A**) the control group, (**B**) the GA group, (**C**) the CMCS group, (**D**) the GA@CMCS group, (**E**) the CDDP group, (**F**) the (CDDP + GA) group, (**G**) the (CDDP + CMCS) group, and (**H**) the (CDDP + GA@CMCS) group. (Caspase-3 expression, scale bar: 50 um). All data have been presented as mean ± SE. *p < 0.05, **p < 0.01, and ***p < 0.001.

In the current investigation, the CDDP-treated rats had a significantly higher level of caspase-3 than the control rats. In comparison to the CDDP group, pre-and post-treatment with GA@CMCS following CDDP injection dramatically lowered caspase-3 levels in both the cortex and the medulla (Fig. 8I(A–H) & II(A–H), respectively). Treatment of rats with CMCS or GA@CMCS for ten continuous days before CDDP injection and additional 4 days after CDDP injection significantly decreased the caspase-3 densitometry immunohistochemistry protein expression as compared with the CDDP group (p < 0.01) and (p < 0.001) respectively. The caspase-3 densitometry immunohistochemistry protein expression was highly considerably increased in the cortex as compared with the medulla as seen in both the CDDP group (p < 0.001) and CMCS group (p < 0.001), also caspase-3 was significantly expressed in the cortex as compared with medulla in (CDDP + GA) group (p < 0.01), (CDDP + CMCS) group (p < 0.05), and (CDDP + GA@CMCS) group (p < 0.05) (Fig. 8III).

### Na^+^/K^+^-ATPase (Sodium potassium adenosine triphosphatase)

**Figure 9:**
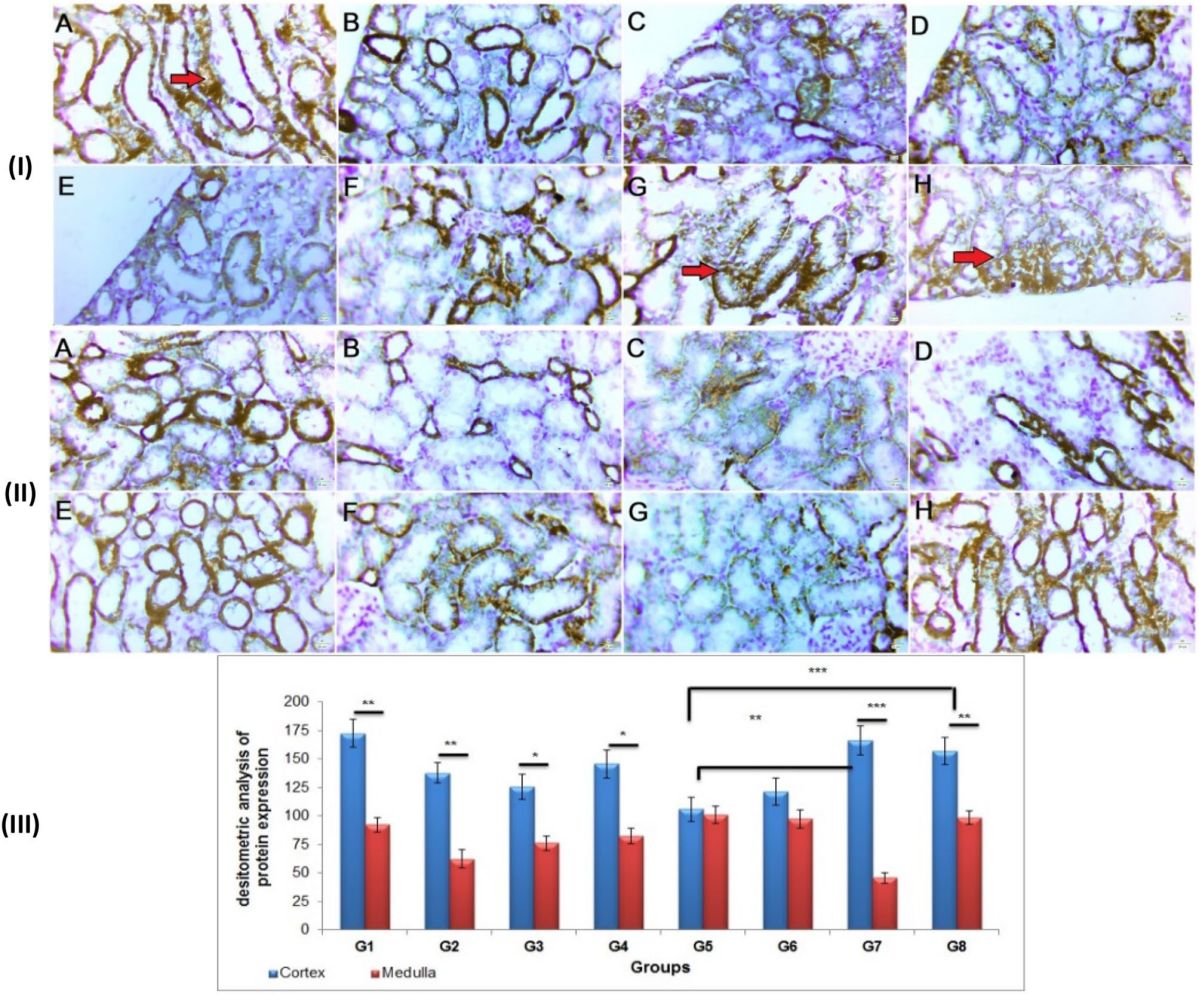
The effect of different treatments on Na^+^/K^+^-ATPase immunoreactivity and densitometry in CDDP-induced nephrotoxicity in all the studied groups. (**I**) a micrograph of a section of the kidney showing Na^+^/K^+^-ATPase immunoreactivity in the cortex. (**II**) a micrograph of a section of the kidney showing Na^+^/K^+^-ATPase immunoreactivity in the medulla. (**III**) Na^+^/K^+^-ATPase densitometry immunohistochemistry protein expression analysis in both cortex and medulla. Micrographs of the kidney section of (**A**) the control group, (**B**) the GA group, (**C**) the CMCS group, (**D**) the GA@CMCS group, (**E**) the CDDP group, (**F**) the (CDDP + GA) group, (**G**) the (CDDP + CMCS) group, and (**H**) the (CDDP + GA@CMCS) group. (Na^+^/K^+^-ATPase expression, scale bar: 50 μm). All data have been presented as mean ± SE. *p < 0.05, **p < 0.01, and ***p < 0.001.

In the current study, i.p injection of CDDP causes nephrotoxicity manifested by a reduction in expression of kidney Na^+^/K^+^-ATPase as compared to the control group. Thus, treatment with CMCS, or GA@CMCS before and after CDDP injection appeared to decrease the harmful effect of CDDP in both cortex and medulla (Fig. 9I(A–H) & II(A–H), respectively). Treatment of rats with CMCS or GA@CMCS for ten continuous days before CDDP injection and additional 4 days after CDDP injection significantly increased the Na^+^/K^+^-ATPase densitometry immunohistochemistry protein expression as compared with the CDDP group (p < 0.01) and (p < 0.001) respectively.

The Na^+^/K^+^-ATPase densitometry immunohistochemistry protein expression was highly considerably increased in the cortex as compared with the medulla as seen in (CDDP + CMCS) group (p < 0.001), also Na^+^/K^+^-ATPase protein was considerably expressed in the cortex as compared with medulla in the control group (p < 0.01), GA group (p < 0.01), CMCS group (p < 0.05), GA@CMCS group (p < 0.05), and (CDDP + GA@CMCS) group (p < 0.01), while there were no significant differences between cortex and medulla in both CDDP group and (CDDP + GA) group (Fig. 9III).

## Discussion

Chemical modifications of CS are generally favored to modify its solubility and capacity to interact with other substances. Carboxymethyl chitosan is a water-soluble biopolymer that exhibits many superior characteristics and potentials (non-toxicity, biocompatibility, biodegradability, and good film-forming capability)^45^. Antioxidant–biopolymer conjugates could be used as new food additives, packaging materials, and biomedical materials to maximize the benefits of each constituent^16^. Notably, GA@CMCS conjugate exhibited a different FT-IR spectrum as compared to its native constituents. For instance, the band observed at 1596 cm^−1^ in the CMCS spectrum was significantly decreased in the GA@CMCS spectrum as well as a new peak has emerged at 1694 cm^−1^, which could be assigned to the stretching of the carbonyl group of amide I. The findings indicate the successful grafting of GA molecules on the surface of CMCS through the covalent linkage between the NH_2_ group of CMCS and the COOH group of GA to form an amide bond. On the other hand, no vibrational band was observed in the spectral region distinctive of the absorption of the ester carbonyl group (1700–1800 cm^−1^)^46^, confirming that no ester bond was formed between the OH groups of the CS backbone and the COOH group of GA. Consequently, the grafting of GA on CMCS occurs only through the amide coupling reaction between NH_2_ and COOH of CMCS and GA, respectively. In UV–Vis spectra, the red-shift of the GA absorption peak from 264.00 to 270.50 nm, could be attributed to the decrease in energy required for the π → π* and n → π* transitions due to the covalent linkage between CMCS and GA, which is indicative of the successful grafting of GA onto CMCS. In addition, the emergence of a new UV peak at 364.00 nm in the spectrum of the GA@CMCS offers further evidence. Another strong proof of the success of GA grafting onto the CMCS surface was provided by the ^1^H NMR spectral technique^.^ As shown in Fig. S2 (ESM S1), GA exhibited only a sharp peak at 6.99 ppm attributable to the resonance of phenyl protons^47^. On the other hand, the two peaks observed at δ 3.86 and 1.83 ppm in the NMR spectrum of LMWCS (Fig. S3, ESM S1) could be assigned to the resonance of protons of the glucopyranose unit and the methyl group of the N-acetyl glucosamine unit, respectively, of the CS backbone^20^. The emergence of a new singlet peak in the spectrum of CMCS (Fig. S4, ESM S1) at δ 4.30 ppm equivalent to two protons of a methylene group, is indicative of the success of chloromethylation of LMWCS to CMCS^42^. As for the ^1^H NMR spectrum of GA@CMCS (Fig. S5, ESM S1), the two sets of signals distinctive to GA and CMCS have been merged. For instance, in addition to the signals of the LMWC backbone, two singlet peaks at δ 4.29 and 6.89 ppm are characteristic of methylene and phenyl protons of CMCS and GA, respectively, can be seen. These findings also confirm the successful grafting of the CMCS surface by GA^44^.

Oxidative stress (OS) has been assigned as one of the primary causal elements in the toxicological consequences of the majority of chemotherapy medicines^10^. As a result of oxidative damage in nephrotoxicity, ROS causes the liberation of H-atoms from unsaturated lipids, resulting in lipid peroxidation. This led to several alterations in the membrane structure and activities of the cell and causes DNA damage, cytotoxicity, and eventually cell death^48^. Our data were consistent with previous work that indicated a significant increase in MDA levels among CDDP-challenged mice^49^. Additionally, the highly increased MDA was due to the CDDP-induced lipid peroxidation and damage of the plasma membrane exerting oxidative stress^48^. Our findings were comparable to the work that was done by Nabavi et al.^50^ which showed that antioxidant pre-treatments might improve NaF-induced nephrotoxicity in rats by direct scavenging of free radicals and/or enhancing the endogenous antioxidant systems. GA showed antioxidant effects through its activity of indirect free radicals scavenging or indirectly by increasing the endogenous antioxidant enzymes' activity and expression^49,51^. In addition, GA@CMCS uptake exerted excellent biological activities in comparison to those of GA or CMCS alone. Further, the acute decrease in the rat's absolute kidney weight and renal index due to cisplatin were improved significantly by the co-treatment with GA@CMCS and attenuation of the cisplatin toxicity that was appeared as distributed shrunken and atrophic features of the cortical tubules with expanded interstitial spaces, and fibrotic glomerulus that resulting from body weight and renal index loss in this study^51,52^.

Additionally, the activity of glutathione (GSH) as a cysteine-rich tripeptide with non-enzymatic antioxidant activity with a reactive thiol group has reductive efficacy by interacting directly with ROS^53^. The reduction in GSH levels caused by CDDP indicates a change in cellular redox state, implying that the cells are more susceptible to ROS^52^. Therefore, our results suggested that the decline in oxidative damage induced by CDDP is due to the antioxidant potential activity of GA@CMCS to improve the body's antioxidant defenses and better than the activity of GA or CMCS, indicating the antioxidant potential role was increased by the conjugation of GA on the CMCS backbone^53^. These findings were harmonized with those of a previous study that reported that grafting GA onto CS using the free radical-mediated conjugation technique increased the biological activities such as antioxidant, antimicrobial, and antibacterial activity. The biological activities of GA-grafted CS were higher than those of unmodified CS^17^. Our findings concurred with previous research results concerning the GA and CS grafting^26,40,48,52^. Based on the fact that gallic acid has therapeutic benefits in reducing oxidative stress and has medicinal characteristics, its low absorption has restricted its application in treatment. Thus the activity of GA@CMCS in the amelioration of the antioxidant system properties came from the carrier bioavailability of O-carboxymethyl chitosan and their potentiality in improving the physical and chemical properties of gallic acid^54,55^. Besides, the nitric oxide (NO) was prominent with a pivotal role in both regulating renal hemodynamic and modulating inflammatory, proliferating responses to various stimuli^26^, and vasodilatation^51^. Inducible nitric oxide synthase (iNOS) synthesizes NO from l-arginine^17^, and its overproduction contributes to OS and tissue damage by interacting with superoxide to produce the deadly agent peroxynitrite^51^. Regarding our study results, Ahn et al.^17^ stated that GA-grafted-CS inhibited significantly lipopolysaccharide (LPS)—stimulated NO and prostaglandin E2 (PGE2) production in macrophages by downregulation of the protein and mRNA expression of iNOS and COX-2. Thus, the restoration of NO levels after treatment of CDDP-intoxicated animals with GA@CMCS may be attributed to the inhibition of iNOS which produces toxic concentrations of NO^56^. Also, the study done by Moradi et al.^40^ discovered a significant decrease in nitrite levels in GA-treated rats, indicating that GA provided nephroprotection in Diclofenac-exposed animals by lowering NO levels and hence nitrosative stress.

Acute kidney injury (AKI) is considered one of the reported serious side-effects of platinum-based chemotherapeutics. However, so far there were no approved biomarkers for detecting the proximal tubular injury. In vitro and in vivo research, KIM-1 has shown to be a promising biomarker in detecting CDDP-induced renal injury^57^. Proximal tubular damage, renal tubular regeneration, and immunological response to nephrotoxicants have all been linked to KIM-1 expression. In the present study, the damaged kidney revealed KIM-1 mRNA and protein levels with extremely high values. KIM-1 has been proposed as a non-invasive biomarker for proximal tubular injury in humans^58^. Our previous study showed that the kidney functions increased significantly in the CDDP group compared to the control group and GA group, as well as, the kidney functions were decreased in the (CDDP + GA) group compared to the CDDP group alone^59^. In the current study, GA@CMCS, which combines the benefits of GA and CMCS, showed a higher ability than GA and CMCS, respectively for minimizing CDDP-induced nephrotoxicity. In prospective research with 123 patients receiving platinum chemotherapeutics, Tanase et al.^57^ reported that urine levels of KIM-1, NGAL, and cystatin C showed a statistically significant elevation on day three after treatment commencement in AKI patients. Additionally, one of the key underlying mechanisms of CDDP-induced nephrotoxicity is DNA damage by blocking DNA replication and cell division restriction leading to apoptosis. The CDDP genotoxicity emanated from its cross-linking with purine bases in DNA, producing DNA damage in malignant cells. The reported DNA damage caused by CDDP treatment in this study was in agreement with Hassan et al.^60^ who found a significant increase in the tail length of DNA, tail intensity (DNA percent), and tail moment among CDDP-treated rats compared to controls to determine the renal genotoxic potential of CDDP. The present study revealed that the conjugation of GA onto CMCS improved the ability of CMCS to prevent DNA damage caused by CDDP and was inconsistent with Boran et al.^61^ who reported that treatment with 50 M CDDP induced significant DNA damage on NRK-52E cells in comparison to the negative control and, both 100 nM and 200 nM celastrol pre-treatment triggered a significant reduction in DNA damage. Moreover, the protective role of GA conjugated onto CMCS may be due to its antioxidant activity in accordance with Wen et al.^62^ and El-Denshary et al.^63^ who reported that chitosan nanoparticles protected cells against H_2_O_2_ and carbon tetrachloride chronic (CCl_4_) toxicity, respectively through its ability to upregulate gene expression of the endogenous antioxidant systems and scavenging free radicals.

The present study data of serum and kidney injuries were coincident with the histopathological analysis. On one side, the morphological and physiological analysis revealed that the renal tubule system is the most sensitive with the highest damage due to the CDDP injection followed by the proximal tubules. On the other side, the administration of antioxidants has been shown to ameliorate CDDP-induced nephrotoxicity in animals^28^. Histopathological findings revealed structural abnormalities in the renal tissue of CDDP-treated rats, which matched the results of the biochemical examination. This proves that the CDDP injection caused the histopathological lesions in the current investigation. The histopathological findings are also consistent with earlier research^37,48,51,53^. Furthermore, Carboxymethyl chitosan revealed its ability to inhibit the tumor growth on hepatocarcinoma 22 cells and decreased tumor cells proliferation with inhibitory rates of 32.63%, 51.43%, and 29.89% at the doses of 75 mg/kg, 150 mg/kg, and 300 mg /kg, respectively^64^.

Moreover, the inducible form COX-2 was involved in the generation of prostaglandins that mediate pain and promote the inflammatory process and are expressed in response to inflammatory and other physiologic stimuli and growth factors. Immunohistochemical findings revealed structural changes in the renal tissue of CDDP-treated rats, which matched with the results of the biochemical assessment and histopathological findings. The protective effects of GA, CMCS, and GA@CMCS against CDDP were confirmed by immunohistochemical studies in the kidney. The treated groups showed significant improvements in proximal and distal convoluted tubules, as well as glomerular atrophy. The number of antioxidant molecules grafted on the backbone of the polymer determines the antioxidant activity of a synthetic product^65^. GA@CMCS, which combines the benefits of both GA and CMCS, produced more antioxidants than either GA or CMCS due to upregulation of its bioavailability and penetration, implying that GA@CMCS can reduce kidney damage and protect the kidney from nephrotoxicity. CDDP, it was discovered, stimulates both the extrinsic and intrinsic apoptotic pathways: the extrinsic pathway, which is initiated by death receptors, and the intrinsic pathway, which is concentrated on the endoplasmic reticulum and mitochondria. CDDP's apoptotic action can be mediated by a p53-dependent or p53-independent response^66^. The major component of apoptosis happens predominantly through a p53-dependent route that involves Bcl-2 family target proteins (i.e., Bax, Bcl-2) and caspase family activation^24^. Caspase-3, an executioner of apoptosis is considered an index of apoptosis^37^. Consistent with previous investigations Abd El-Rhman et al.^67^ stated that the CDDP-treated rats had a 483.7 percent rise in caspase-3 levels when compared to the control rats. In comparison to the CDDP group, pretreatment of CDDP-injected rats with Dibenzazepine dramatically lowered caspase-3 levels by 43.2%.

Furthermore, the present study revealed that the gallic acid, CMCS, and GA@CMCS significantly decreased the caspase-3 immunoexpression among CDDP injected rats may be due to its ability to inhibit the toxicity of its metabolites [Pt(NH_3_)_2_(H_2_O)_2_]^2 +^ and [Pt(NH_3_)_2_Cl(H_2_O)]^+^. Our data were in accordance with Fang et al.^68^ who concluded that chitosan (COS) administration exerted anti-oxidative effects through activating superoxide dismutase and catalase, leading to decreased renal apoptosis and reduced renal NF-κB p65. An in vitro study demonstrated COS increased IκB expression, attenuated the increase of p65, and thus decreased NF-κB/DNA binding activity in PQ-stimulated RGC-5 cells. In conclusion, COS attenuates oxidative stress-induced renal damages, probably by decreasing free radicals, maintaining the activities of anti-oxidative enzymes, and inhibiting the activation of NF-κB and in disagree with Ji et al.^69^ who reported that GA induced apoptosis in NCI-H460 lung cancer cells via a caspase-3 and inhibited the in vivo tumor growth of NCI-H460 cells in xenograft models. Furthermore, CDDP therapy can change the expression of Na^+^/K^+^-ATPase. Na^+^/K^+^-ATPase is an enzyme found in the membranes of practically all animal cells. It is very important in cell physiology. For each ATP molecule consumed by the pump, three sodium ions are withdrawn and two potassium ions are imported, resulting in a single positive charge being exported every pump cycle. Furthermore, decreased Na^+^/K^+^-ATPase activity may be attributed to lower amounts of its substrate (ATP), and increased membrane lipid peroxidation leads to membrane modification by ROS^70^. Therefore, the unregulated expression levels of Na–K-ATPase not only depend upon its functions as a physiological ion transporter, but it also functions as a signaling transducer leading to the generation of ROS and oxidative modification of the protein. Our results agreed with that of Alazragi^70^ which revealed that rats were exposed to Amiodarone A when compared to the control group, supplementation produces pulmonary toxicity, as evidenced by a significant drop in serum value of lung Na^+^/K^+^-ATPase. Thus, using either ferulic acid or GA or a combination of the two reduced Amiodarone A harmful effects.

## Conclusion

Our data showed that GA@CMCS has a protective effect in vivo against CDDP-induced nephrotoxicity by lowering excessive apoptosis, oxidative stress, and inflammation. COX-2 densitometry immunohistochemistry expression was dramatically reduced in rats treated with CMCS or GA@CMCS pre and post CDDP administration. Caspase-3 densitometry immunohistochemistry protein expression was dramatically reduced in rats given CMCS or GA@CMCS before or after CDDP administration. The Na^+^/K^+^-ATPase densitometry immunohistochemistry protein expression was considerably elevated in rats treated with CMCS or GA@CMCS before or after CDDP injection. As a result, it will be a fascinating topic to investigate further in the future. Finally, our study revealed the protective role of gallic acid and gallic acid conjugated onto carboxylic chitosan among cancer patients treated with cisplatin after their therapy doses to impair or hinder cisplatin's toxicity on other body organs with the maintenance of the cisplatin activity during chemotherapy. In addition, further research is needed to shed light on the mechanistic roles of the prepared compounds in the diminution of the cisplatin toxicity and how the novel synthesized compounds trigger or inhibit the proinflammatory and inflammatory cytokines expression.

## Supplementary Information

Supplementary Information.

## Data Availability

The datasets generated and/or analyzed during the current study are available from the corresponding author on reasonable request.
