# Supplementary material for: The diminution and modulation role of water-soluble gallic acid-carboxymethyl chitosan conjugates against the induced nephrotoxicity with cisplatin
Source: Sci Rep. 2022 Nov 19;12:19903. doi: 10.1038/s41598-022-21681-8 (PMC9675851; doi:10.1038/s41598-022-21681-8)
Supplement: Supplementary file 1 — Supplementary Information. [file 41598_2022_21681_MOESM1_ESM.pdf]

The diminution and modulation role of water-soluble gallic acid-carboxymethyl chitosan conjugates against the induced nephrotoxicity with cisplatin

Hani S. Hafez[1], Ebtesam S. Kotb[2], Zakaria El-Khayat[3], Reda F.M. Elshaarawy[2,4] & Waleed M. Serag[2]

[1] Zoology department, Faculty of Science, Suez University, 43533 Suez, Egypt.

[2] Chemistry Department, Faculty of Science, Suez University, 43533 Suez, Egypt.

[3] Medical biochemistry department, National Research Center Egypt

[4] Institut für Anorganische Chemie und Strukturchemie, Heinrich-Heine Universität Düsseldorf, Düsseldorf, Germany.

Corresponding author: RFME Email: reda.elshaarawy@suezuniv.edu.eg; reel-001@hhu.de.

Contents:

Materials and methods
Synthesis and characterization of CS and LMWC
Figures

Materials and method

Materials, Chemicals were obtained from the following suppliers and used without further purification: dodecyl sodium sulfate (SDS), monochloroacetic acid , tetraisopropyl orthotitanate (TIPT), acetylacetone (AcAc) (Sigma–Aldrich).

Instrumentation, Elemental analyses for C, H, N and S were performed with a Perkin–Elmer 263 elemental analyzer. FT-IR spectra were recorded on a BRUKER Tensor-37 FT-IR spectrophotometer in the range 400–4000 cm^{-1} as KBr discs or in the 4000-550 cm^{-1} region with 2 cm^{-1} resolution with an ATR (attenuated total reflection) unit (Platinum ATR-QL, Diamond). For signal intensities the following abbreviations were used: br (broad), sh (sharp), w (weak), m (medium), s (strong), vs (very strong). NMR-spectra were obtained with a Bruker Avance DRX200 (200 MHz for ^1H) or Bruker Avance DRX500 (500 MHz for ^{13}C) spectrometer with calibration to the residual proton solvent signal in D_2O (^1H NMR: 4.79 ppm) against TMS with $\delta = 0.00$ ppm. Multiplicities of the signals were specified s (singlet), d (doublet), t (triplet), q (quartet) or m (multiplet). The particle shape of new materials was examined using transmission electron microscope (TEM). The images were taken by a JEM-2011F microscope (JEOL, Japan) operated at 200 kV. The morphology of the formed micro and nano-composites was investigated using Scanning electron microscopy (SEM, Hitachi S-7400, Hitachi, Japan) supported with energy dispersive –X-ray (EDX) to determine the elemental analysis of the formed products..

Synthesis and characterization of CS and LMWCS

Synthesis of chitosan (CS), The crabs were obtained from Bay of Suez coast. The terminals and operculum of crabs were removed and the shells (~1500 g) are scraped free of loose tissue and washed individually in lightly saline water, then separated from cephalothoraxes, salted (5 kg of NaCl per 250 g of shells), washed thoroughly in distilled water and placed in Ziploc bags and refrigerated overnight. The crab's exoskeletons were crushed into smaller pieces using a meat tenderizer and dried in the sun (25-30 °C) for 3 days, and finally oven-dried for a week at 65°C until constant weight. After that, the dried shells were grinded, sieved, and the fraction below 80

μm was used hereafter. The extraction method proposed here involved three chemical treatment steps, with each step followed by rinsing with distilled water until a neutral pH was reached. In the first step (demineralization process), 100 g of shrimp shells powder was immersed in 1000 mL of 0.5 M HCl at ambient temperature (25 °C) under constant stirring for 24 h. After washing with distilled water, the second step of the procedure was the deproteination stage in which the residue was immersed in 1000 mL of 1 M NaOH under vigorous stirring at 60 °C for 24 h. Then the proteins were removed by filtration. Distilled water was used to wash the residue to neutral. Then, the residue was subjected to the above procedure two more times. The chitin obtained still had a slight pink colour. Further decolourisation was achieved by soaking chitin in 250 mL of 1% $KMnO_4$ for 1 h. followed by 250 mL of 1% oxalic acid for 2 h. The amount of 250 mL of 95% ethanol and 200 mL of absolute ethanol were sequentially used to remove ethanol-soluble substances from the obtained crude chitin and to dehydrate the chitin. Finally the chitin was dried in air at 50 °C overnight. Yield 86.34 g (86.34% based on 100 g of crab shell powder). The final step is the deacetylation process in which the purified chitin was deacetylated to form chitosan by treating 10 g of chitin with 100 mL 2 M NaOH under stirring at 60 °C for 72 h. After filtration, the residue was washed three times with hot deionized water at 60 °C. The crude chitosan (7.9 g) was obtained by drying in an air oven at 50 °C overnight. The obtained crude chitosan was purified by dissolution in 1% (v/v) aqueous acetic acid until a homogenous solution is obtained, filtered through 22 μm Whatman filter paper to remove insoluble impurities, then precipitated by titration with 1 M NaOH until pH value of 8.5, and finally washed several times with distilled water. Yield 6.8 g (89 % based on chitin). FTIR (KBr, cm^{-1}): 3438 (m, br), 3186 (m, br), 1654 (vs, sh), 1572 (m, sh), 1380 (m, sh), 1069 (m, sh), 895 (m, sh). ^{1}H NMR (600 MHz, 1% $CD_3COOD/D_2O)_{60\,°C}$ δ (ppm): 5.48 (d, *J* = 7.2 Hz, 1H), 4.81– 4.73 (m, 8H), 4.11-3.89 (m, 3H), 3.54 (s, 1H), 2.43 (s, 3H). Degree of acetylation 14.91%. Anal. Calcd. for $(C_8H_{13}NO_5)_{0.149}(C_6H_{11}NO_4)_{0.851}.H_2O$ (M = 185.44 g/mol) : C, 40.79; H, 7.23; N, 7.55; Found C, 40.68; H, 7.32; N, 7.31.

Deacetylation of CS, A solution of CS (4.0 g) in aqueous acetic acid (200 mL, 2 wt%) was added dropwise to aqueous NaOH (50%, 100 mL) at room temperature under magnetic stirring and in an atmosphere of nitrogen. At the end of the addition, the suspension was refluxed for 1 h. It was poured into stirred water (4 L) preheated to 80 °C. The precipitate was decanted, washed five times with water until neutral pH, and separated by filtration. The resulting polymer was purified by dialysis against water for 3 days and isolated by lyophilization (yield 3.28 g, 82%). The DDA determined by ^{1}H NMR was 95.32%. (600 MHz, 1% $CD_3COOD/D_2O)_{60\,°C}$ δ (ppm): 5.02 (d, *J* = 7.1 Hz, 1H), 4.41– 4.18 (m, 6H), 4.02-3.89 (m, 3H), 3.38 (s, 1H), 2.39 (s, 3H).

Preparation of low molecular weight chitosan (**LMWCS**), Chitosan solution (2%) was prepared in 1% CH_3COOH by stirring for 24 h in room temperature. A solution of H_2O_2 30% (4.4%) was then used to achieve chemical degradation of chitosan in 30 for 1.5 h. Adjusting the pH of the solution to approximately 7 was carried out by using 1 M NaOH solution. As the pH increased, part of chitosan was precipitated. Thereafter, the solution was centrifuged at 6000 rpm for 30 minutes to separate sediments. The upper phase of the centrifuged solution included water-soluble chitosan (WSCS), which was soluble in neutral pH, and the lower phase consisted of low molecular weight chitosan (LMWCS). Then, WSCS was vacuum filtered with the aid of appropriate filter paper, while the wet LMWC was submitted to ultrasonic irradiation with an amplitude of 100 Hz for 20 minutes in order to break the chains further. The final product was dried under vacuum at 40 °C for 48 h and chracterized based upon FTIR, ^{1}H NMR and molecular weight (MW) determination by viscometric

measurements using an Ubbelohde Capillary Viscometer (0.5 mm). Average molecular weights were calculated from $[\eta] = k.M^{\alpha}$ equation, where η intrinsic viscosity, $k = 1.81 \times 10^{3}$ (cm^3/g) and $\alpha = 0.93$ determined in 0.25M CH_3COONa and 0.25M CH_3COOH solution at 25 °C.

Structural characterizations of LMWCS

Molecular weight, microanalytical data, degree of acetylation (DA) The molecular weights of CS and LMWC samples can be calculated based upon their intrinsic viscosities (η) according to the Mark–Houwink–Sakurada (MHS) (502.76 and 21.83 mL/g, respectively) and were found to be 713.6 and 24.5 kDa, respectively. As shown, the molecular weight of LMWC system was decreased by ~97% compared to the native CS. As the LMWCS was obtained by partial deacetylation of chitin followed by partial degradation of CS, so the proposed empirical formula for LMWCS is $(\beta\text{-D-Glc-NHAc})_{DA}(\beta\text{-D-Glc-NH}_2)_{100\text{-}DA}$. Elemental analysis (EA) provides a powerful tool for calculation the degree of acetylation (DA) calculated according to the following equation.

$$DA = [(C/N - 5.14)/1.72] \times 100\%$$

Based on the elemental analysis and degree of deacetylation, we propose a an average "repeat or building unit" of LMWC as $(GlcNHAc)_{0.045}(GlcNH_2)_{0.955}$ or $(C_8H_{13}NO_5)_{0.242}(C_6H_{11}NO_4)_{0.758}(H_2O)$ (M = 189.34 g/mol). ^1H NMR of LMWC is depicted in Fig. S1.

Determination of degree of carboxymethylation (DC)

The DC of CMCS derivatives was determined by dissolving CMCS derivative (0.3 g) in 0.1 mol L^{-1} HCl (30 mL) and titrating with 0.1 mol l^{-1} aqueous NaOH. The DC value was calculated as follows [S1]:

$$DC = \frac{161\ A}{m_{CMCS} - 58\ A}$$

$$A = V_{NaOH} - C_{NaOH},$$

where V_{NaOH} and C_{NaOH} were the volume and molarity of aqueous NaOH, respectively, mCMCS was the mass of CMCS (g), and 161 and 58 are the molecular weights of the glucosamine (chitosan skeleton unit) and the carboxymethyl group, respectively.

Determination of grafting degree of GA@CMCS

The measurement of GA content in GA@CMCS was carried out according to the Folin– Ciocalteu method with slight modification.[1] Briefly, the freeze-dried GA@CMCS powder was dissolved in deionized water at 1 mg/mL. Then, 0.5 mL of each sample was mixed with 1 mL of Folin–Ciocalteu reagent (10 times dilution) and reacted for 5 min in the dark, followed by the addition of 2 mL 15% Na_2CO_3 and deionized water till 10 mL. The mixture was shaken and maintained under room temperature for 2 h and the absorbance of the reaction mixture was read at 750 nm using UV-vis spectrophotometer (Evolution 201, Thermo Fisher Scientific Inc., Waltham, MA, USA). GA was used as a standard and the grafting degrees of GA@CMCS were expressed as mg of GA equivalents per g of GA-CS (or weight percent).

Figures Captions

Figure S1. Mechanism of action of CDDP including (i) cellular uptake, (ii) aquation/activation, (iii) Platination of DNA, and (iv) cellular processing leading to apoptosis

Figure S2. ^{1}H NMR spectrum of GA (400 MHz, D_2O).

Figure S3. ^{1}H NMR spectrum of LMW**C** (400 MHz, D_2O).

Figure S4. ^{1}H NMR spectrum of *O*-CMLMW**C** (400 MHz, D_2O).

Figure S5. ^{1}H NMR spectrum of GA@CMLMW**C** (400 MHz, D_2O).

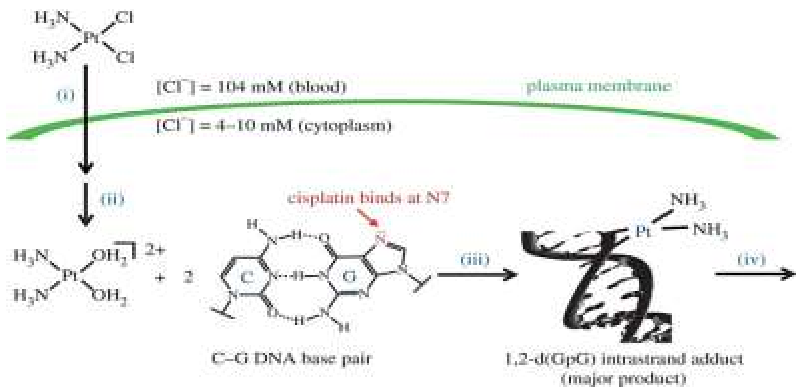

Figure S1. Mechanism of action of CDDP including (i) cellular uptake, (ii) aquation/activation, (iii) Platination of DNA, and (iv) cellular processing leading to apoptosis.

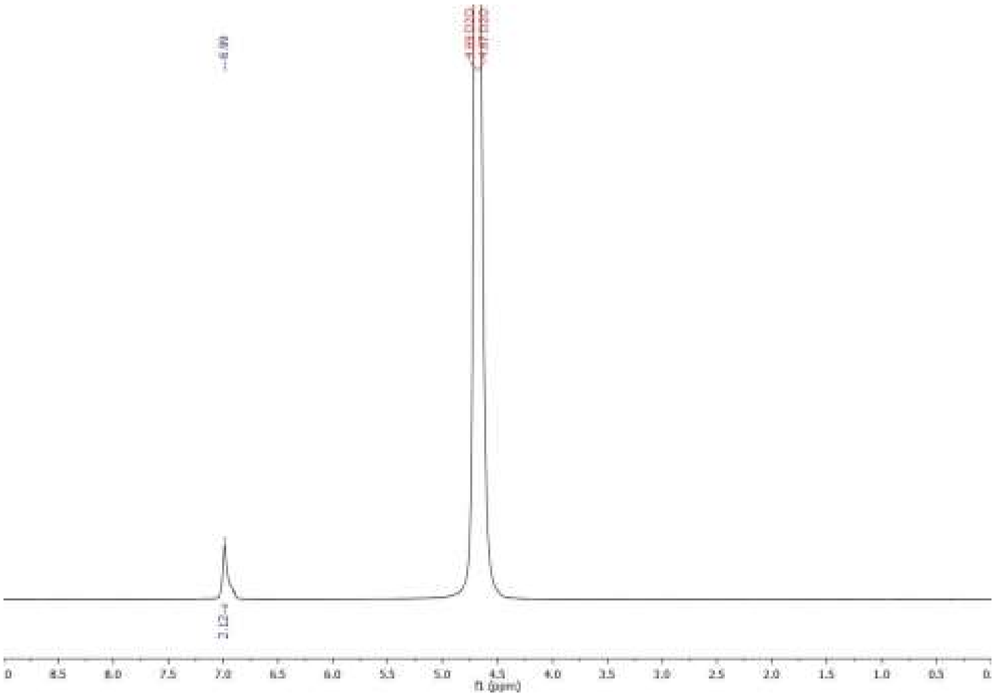

Figure S2. ^{1}H NMR spectrum of GA (400 MHz, D_2O)

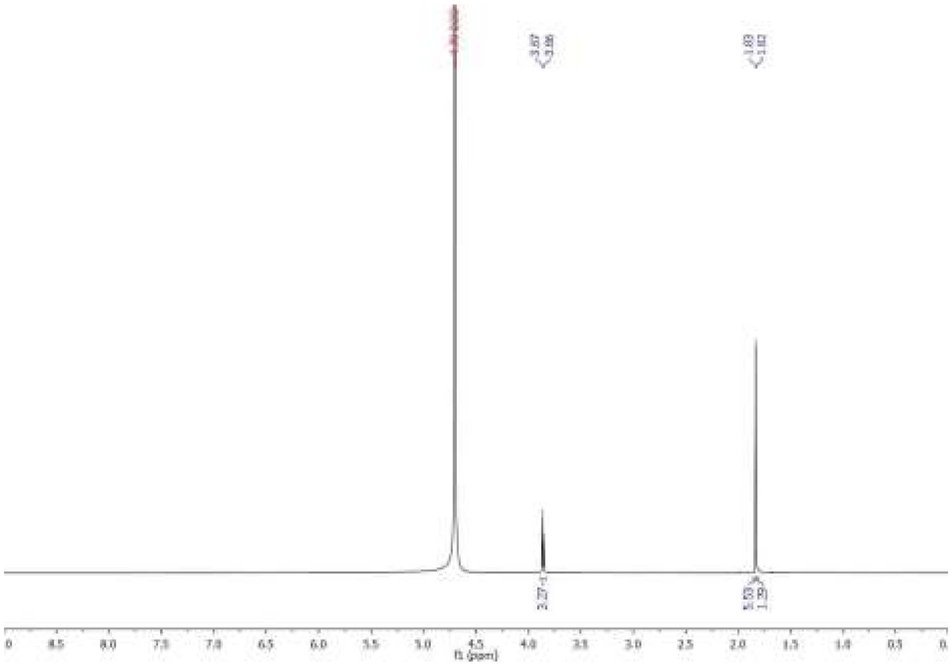

Figure S3: ^{1}H NMR spectrum of LMW**C** (400 MHz, D_2O).

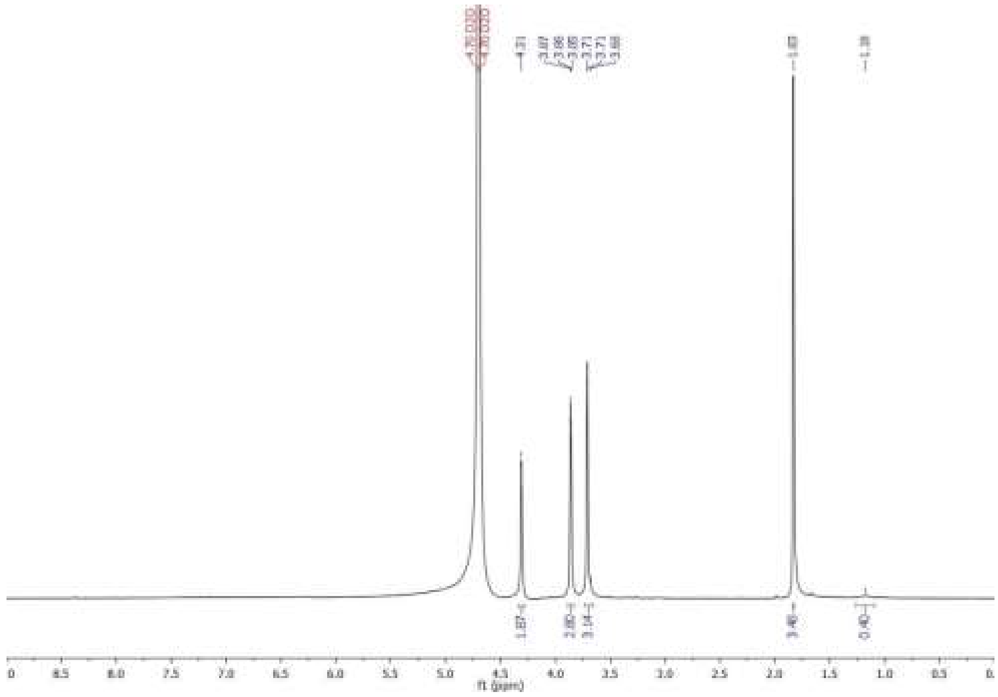

Figure S4. ^{1}H NMR spectrum of *O*-CMLMWC (400 MHz, D_2O)

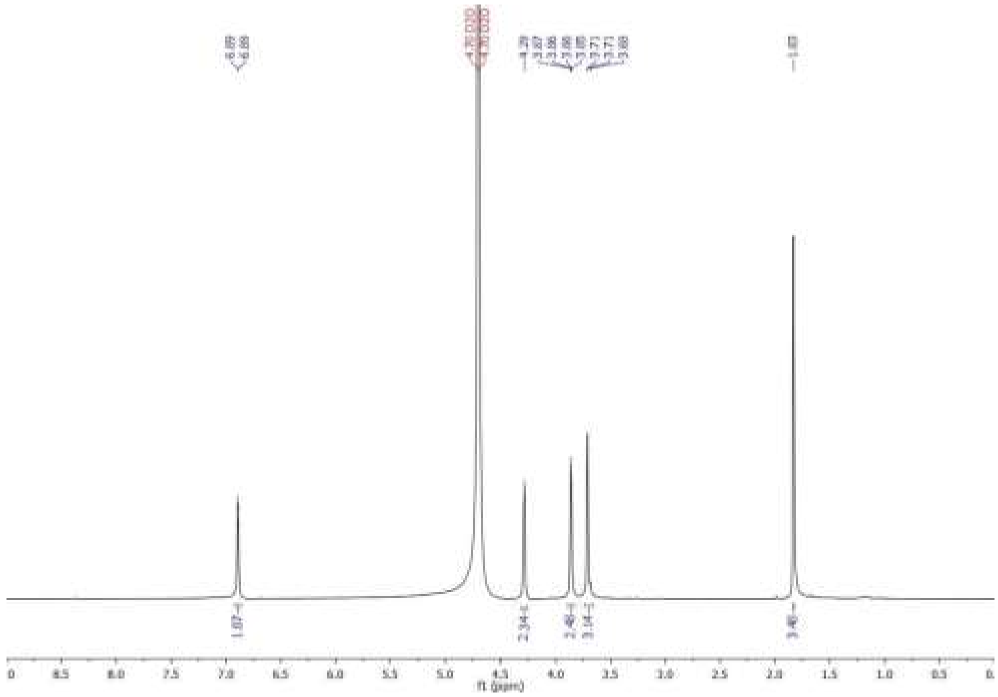

Figure S5. ^{1}H NMR spectrum of GA@CMLMWC (400 MHz, D_2O)

References

S1 R. Muzzarelli, F. Tanfani, M. Emanuelli, Carbohydr. Res. 126 (1984) 225
